# Supplementary material for: Leveraging ONT move table values for signal aware variant calling
Source: Bioinformatics. 2026 Aug 21;42(Suppl 2):btag376. doi: 10.1093/bioinformatics/btag376 (PMC13501324; doi:10.1093/bioinformatics/btag376)
Supplement: Supplementary file 1 [file btag376_supplementary_data.pdf]

Supplementary Materials

Additional Methods and Results for Signal-Aware Variant Calling with ONT Move Tables

Table of Contents

Data processing procedures **2**
Basecalling and alignment 2
Variant calling commands 2
Benchmarking commands 3

Model details **4**
Architecture 4
Description of DNA pileup input features 4
Description of DNA full-alignment input features 5
Description of network outputs 6
Training configurations 6

Supplementary tables **7**

Supplementary figures **16**

Data processing procedures

Basecalling and alignment

Raw POD5 files were basecalled using Dorado with R10.4.1 400 bps SUP and HAC models. The human reference genome (GRCh38 no-alt analysis set) was provided during basecalling to enable reference-guided alignment. The `--emit-moves` option was enabled to output move-table information, which records the mapping between raw signal events and basecalled nucleotides. The resulting BAM files were used for downstream signal-aware variant calling experiments.

For SUP basecalling:

```
dorado basecaller dna_r10.4.1_e8.2_400bps_sup@v5.2.0 pod5_dir/ \
  --reference ref.fa \
  --emit-moves \
  --models-directory model_dir/ \
  > output.bam
```

For HAC basecalling:

```
dorado basecaller dna_r10.4.1_e8.2_400bps_hac@v5.2.0 pod5_dir/ \
  --reference ref.fa \
  --emit-moves \
  --models-directory model_dir/ \
  > output.bam
```

Variant calling commands

Clair3

```
python3 run_clair3.py \
  --bam_fn path_to_bam_file \
  --ref_fn GRCh38_no_alt_analysis_set.fasta \
  --platform ont \
  --model_path path_to_model_directory \
  --threads 60 \
  --output clair3_output \
  --sample_name SAMPLE \
  --enable_dwell_time
```

DeepVariant

```
singularity run deepvariant_1.9.0.sif \
  /opt/deepvariant/bin/run_deepvariant \
  --model_type ONT_R104 \
  --ref GRCh38_no_alt_analysis_set.fasta \
  --reads path_to_bam_file \
  --output_vcf deepvariant_output.vcf.gz \
  --output_gvcf deepvariant_output.g.vcf.gz \
  --num_shards 60
```

Dorado Variant

```
dorado variant \
  --threads 16 \
  --infer-threads 2 \
  path_to_bam_file \
  GRCh38_no_alt_analysis_set.fasta \
  --model path_to_variant_model \
  > dorado_variant_output.vcf
```

LongcallD

```
longcallD call \
  -t 60 \
  GRCh38_no_alt_analysis_set.fasta \
  path_to_bam_file \
  --ont \
  -n SAMPLE \
  > longcallD_output.vcf
```

Benchmarking commands

Variant calling performance was assessed using hap.py, qfy.py, and RTG Tools vcfeval. Predicted VCFs were compared against Genome in a Bottle high-confidence truth callsets using haplotype-aware matching with the GRCh38 reference, and, unless otherwise stated, restricted to GIAB confident regions. hap.py was run with the vcfeval engine and QUAL-based ROC generation, while qfy.py was applied to hap.py outputs to produce stratified performance reports across predefined genomic categories. In parallel, RTG Tools vcfeval was used to independently benchmark query VCFs against truth VCFs using reference SDFs. Precision, recall, and F1-score were reported for SNPs and small indels, and in selected analyses further stratified by zygosity.

hap.py

```
hap.py \
  path_to_truth.vcf.gz \
  path_to_query.vcf.gz \
  -o happy \
  -r GRCh38_no_alt_analysis_set.fasta \
  -f path_to_confident_regions.bed \
  --threads 70 \
  --roc QUAL \
  --engine=vcfeval \
  --engine-vcfeval-template path_to_reference_sdf
```

qfy.py

```
qfy.py \
  happy.vcf.gz \
  -t ga4gh \
  --stratification GRCh38-all-stratifications.tsv \
```

```
  -o strat/report \
  -r GRCh38_no_alt_analysis_set.fasta \
  --threads 60
```

rtg tools vcfeval

```
rtg vcfeval \
  -b path_to_truth.vcf.gz \
  -c path_to_query.vcf.gz \
  -t path_to_reference_sdf \
  --region chr20 \
  --evaluation-regions path_to_confident_regions.bed \
  --output-mode split \
  --roc-subset snp,non-snp,indel,hom,het \
  -f QUAL \
  --threads 32 \
  -o rtg_eval_output_dir
```

Model details

Architecture

For the pileup model, the pileup tensor is processed sequentially using a bidirectional recurrent architecture. Two stacked bidirectional LSTM layers with hidden sizes of 128 and 160, respectively, are used to model long-range dependencies across the genomic window.The output of the recurrent layers is flattened and passed through a dropout layer (dropout rate 0.2) before branching into two task-specific fully connected heads. Each head consists of a dense layer with 128 units, predicting (i) the genotype class and (ii) the zygosity state.

For the full-alignment model, feature extraction is performed using a deep convolutional neural network composed of multiple convolutional blocks. The network applies 3 × 3 convolutions with increasing channel depth (64, 128, and 256), interleaved with ResBlocks to stabilize training and improve gradient flow. Strided convolutions (stride = 2) are used to progressively reduce spatial resolution while expanding the feature dimension. To aggregate spatial information across varying read depths and local contexts, the network incorporates a spatial pyramid pooling (SPP) layer, which pools feature maps at multiple spatial scales (e.g., 1 × 1, 2 × 2, and 3 × 3).After pyramid pooling, features are flattened and regularized using dropout (dropout rate 0.2), followed by a dense layer with 256 units. The model then branches into four parallel prediction heads, each implemented as a dense layer with 128 units, to predict genotype, zygosity, and two indel length–related outputs. Additional dropout (0.5 and 0.2) is applied in the prediction heads to mitigate overfitting.

Description of DNA pileup input features

The pileup tensor has shape [33, 18], representing a 33-position window (flankingBaseNum=16) centered on the candidate variant. The 18 feature channels encode strand-specific nucleotide counts and indel information: A, C, G, T, I, I1, D, D1, *, a, c, g, t, i,

i1, d, d1, #. Channels follow a strand-specific encoding scheme where uppercase letters represent one strand and lowercase letters represent the complementary strand:

1. Base count channels (A/a, C/c, G/g, T/t): Strand-specific counts of each nucleotide at each position. These channels capture the primary sequence information and strand bias patterns.
2. Insertion channels (I/i, I1/i1): I/i channels represent the total insertion support at each position (summed across all insertion variants), while I1/i1 channels specifically count the most frequently observed insertion variant. This dual representation allows the model to distinguish between positions with a single dominant insertion versus positions with multiple competing insertions.
3. Deletion channels (D/d, D1/d1): D/d channels represent the total support for deletions starting at each position, while D1/d1 channels count only the most frequently observed deletion. Similar to insertions, this enables detection of deletion variants and assessment of their complexity.
4. Deletion continuation channels (*, #): These channels mark positions that are within a deletion span but not the deletion start position. This allows the model to distinguish between deletion-start positions and deletion-continuation positions.
5. Reference encoding: Reference base channels are encoded using a signed representation where the reference base position is overwritten with the negative sum of the opposite strand's base counts. This encoding explicitly marks the reference base while preserving strand-specific information.

Description of DNA full-alignment input features

The full-alignment tensor has shape [89, 33, C] where 89 represents the maximum read depth for ONT data, 33 represents genomic positions in the window around the candidate variant, and C is the number of feature channels (8 in standard mode, 9 when dwell-time is enabled). Reads are sorted by haplotype tag and read order before tensor construction. When coverage exceeds 89 reads, excess reads are downsampled to maintain the fixed tensor dimensions.

1. Reference base: Encoded representation of the reference genome base at each position (A, C, G, T mapped to fixed numeric codes).
2. Alternative base: Encodes the read base at each position, specifically highlighting mismatches and indel types relative to the reference. This channel captures variant evidence at the read level.
3. Strand information: Binary encoding indicating whether each read is mapped to the forward or reverse strand, enabling detection of strand-specific artifacts and true strand bias associated with variants.
4. Mapping quality: Per-read mapping quality normalized to the range [0, 100], with values capped at MQ=60. This channel helps the model downweight poorly aligned reads.
5. Base quality: Per-base quality score normalized to [0, 100], with values capped at BQ=40. This provides position-specific confidence in the sequenced nucleotide.
6. Variant support: Normalized per-read allele frequency support for the candidate variant, scaled to [0, 100]. This channel explicitly marks which reads support the variant versus the reference allele.

7. Inserted bases: Encodes insertion sequences by projecting the inserted bases across subsequent positions in the read. This representation allows the convolutional layers to process insertion content spatially.
8. Phasing information: Encodes haplotype assignments (haplotype 1, haplotype 2, or unphased) derived from BAM HP tags. This enables the model to leverage phasing information when available, improving accuracy for compound heterozygous variants and linked polymorphisms.
9. Dwelling time (enabled by `--enable_dwell_time`): This channel is extracted from the ONT BAM mv tag (move table) and aligned to read positions. Dwell-time features provide nanopore-specific signal-level information that can improve variant discrimination, particularly for homopolymers and low-complexity regions where base-calling uncertainty is high.

Description of network outputs

Clair3 is formulated as a multi-task classification problem with up to four prediction heads, enabling simultaneous prediction of variant genotype, variant type, and structural characteristics:

- **GT21 head (21 classes):** Predicts the specific genotype combination from 21 possible classes: AA, AC, AG, AT, CC, CG, CT, GG, GT, TT (SNV genotypes), DelDel, ADel, CDel, GDel, TDel (deletion genotypes), InsIns, AIns, CIns, GIns, TIns (insertion genotypes), and InsDel (compound indel). This fine-grained classification enables precise variant representation.
- **Genotype class head (3 classes):** Classifies variants into three zygosity states: 0/0 (homozygous reference), 1/1 (homozygous variant), and 0/1 (heterozygous variant, which includes both 0/1 and 1/2 genotypes merged into a single heterozygous class).
- **Indel length heads (2× 33 classes):** For the full-alignment model, two additional heads predict the length of insertions or deletions for each allele. Each head outputs a 33 class softmax distribution representing indel lengths from -16 (16 bp deletion) to +16 (16 bp insertion), with lengths clipped to this range.

Training configurations

Models were trained using distributed data-parallel execution across four GPUs. Optimization was performed with the AdamW optimizer and a base learning rate of 1×10^{-4}. Learning-rate scaling was enabled, such that the effective learning rate was multiplied by the number of GPUs; with four GPUs, this resulted in an effective learning rate of 4×10^{-4}.

Training was conducted for a maximum of 30 epochs. Early stopping was applied based on validation loss, with a patience of 10 epochs. The checkpoint corresponding to the lowest validation loss was retained for downstream evaluation.

A random split of the training data was used to form training and validation sets, with 90% of samples used for training and 10% for validation. The loss function consisted of per-head focal loss (focusing parameter $\gamma = 2$), summed across all active prediction heads. L2 weight decay for full-alignment training was set to 1×10^{-7}.

Supplementary tables

Supplementary table 1 *Clair3 v2 (HAC) performance*

Type	Sample	TRUTH.TOTAL	Depth	TRUTH.TP	TRUTH.FN	QUERY.TOTAL	QUERY.FP	QUERY.UNK	FP.gt	FP.al	METRIC.Recall	METRIC.Precision	METRIC.F1_Score
SNP	HG001	3,254,386	10x	3,138,888	115,498	4,085,979	41,887	904,602	26,189	3,572	96.45%	98.68%	97.55%
			20x	3,232,222	22,164	4,279,492	9,420	1,036,931	1,982	1,684	99.32%	99.71%	99.51%
			30x	3,240,024	14,362	4,378,287	6,074	1,131,143	863	1,102	99.56%	99.81%	99.69%
			40x	3,244,675	9,711	4,435,819	5,362	1,184,632	724	862	99.70%	99.84%	99.77%
			50x	3,247,795	6,591	4,484,603	4,562	1,231,073	710	720	99.80%	99.86%	99.83%
	HG003	3,327,495	10x	3,218,821	108,674	4,144,864	39,226	885,923	24,157	3,879	96.73%	98.80%	97.75%
			20x	3,297,399	30,096	4,343,070	10,541	1,033,832	2,540	2,214	99.10%	99.68%	99.39%
			30x	3,304,347	23,148	4,443,503	7,171	1,130,507	1,384	1,398	99.30%	99.78%	99.54%
			40x	3,309,611	17,884	4,492,382	6,222	1,175,022	1,203	1,161	99.46%	99.81%	99.64%
			50x	3,313,946	13,549	4,537,053	5,888	1,215,635	1,281	1,061	99.59%	99.82%	99.71%
	HG004	3,346,610	10x	3,225,924	120,686	4,144,406	40,469	877,052	27,463	3,805	96.39%	98.76%	97.56%
			20x	3,315,702	30,908	4,360,400	8,849	1,034,419	2,525	1,863	99.08%	99.73%	99.40%
			30x	3,324,269	22,341	4,460,165	6,141	1,128,217	1,300	1,444	99.33%	99.82%	99.57%
			40x	3,330,232	16,378	4,514,894	5,181	1,177,901	1,272	1,208	99.51%	99.84%	99.68%
			50x	3,334,931	11,679	4,563,123	4,120	1,222,428	1,167	1,054	99.65%	99.88%	99.76%
	HG005	3,275,631	10x	3,170,659	104,972	4,113,268	39,149	902,690	23,492	3,516	96.80%	98.78%	97.78%
			20x	3,252,004	23,627	4,306,863	8,555	1,045,183	2,368	1,497	99.28%	99.74%	99.51%
			30x	3,260,071	15,560	4,420,498	6,120	1,153,037	1,003	1,052	99.53%	99.81%	99.67%
			40x	3,264,268	11,363	4,475,368	5,499	1,204,291	839	910	99.65%	99.83%	99.74%
			50x	3,267,558	8,073	4,521,883	4,450	1,248,537	821	801	99.75%	99.86%	99.81%
	HG006	3,269,860	10x	3,165,484	104,376	4,072,592	39,430	867,195	24,506	2,881	96.81%	98.77%	97.78%
			20x	3,244,483	25,377	4,264,902	9,141	1,010,576	2,563	1,226	99.22%	99.72%	99.47%
			30x	3,251,550	18,310	4,370,470	6,382	1,111,738	1,207	869	99.44%	99.80%	99.62%
			40x	3,256,211	13,649	4,442,085	6,411	1,178,644	1,169	797	99.58%	99.80%	99.69%
			50x	3,260,086	9,774	4,487,962	5,506	1,221,540	1,080	680	99.70%	99.83%	99.77%
	HG007	3,284,462	10x	3,173,896	110,566	4,090,800	39,029	877,408	26,354	2,696	96.63%	98.79%	97.70%
			20x	3,252,281	32,181	4,279,621	8,530	1,018,119	2,923	1,246	99.02%	99.74%	99.38%
			30x	3,260,661	23,801	4,368,895	5,622	1,101,857	1,237	870	99.28%	99.83%	99.55%
			40x	3,265,598	18,864	4,429,877	4,788	1,158,700	1,094	726	99.43%	99.85%	99.64%
			50x	3,271,127	13,335	4,489,217	4,613	1,212,684	1,075	747	99.59%	99.86%	99.73%
INDEL	HG001	467,702	10x	294,222	173,480	527,091	28,829	198,324	11,982	9,569	62.91%	91.23%	74.47%
			20x	351,018	116,684	632,251	24,325	248,644	8,917	10,794	75.05%	93.66%	83.33%
			30x	372,097	95,605	680,950	24,156	275,308	8,575	11,645	79.56%	94.05%	86.20%
			40x	384,154	83,548	711,806	24,281	293,251	8,666	12,137	82.14%	94.20%	87.76%
			50x	391,619	76,083	732,041	24,305	305,484	8,756	12,493	83.73%	94.30%	88.70%
	HG003	504,501	10x	317,355	187,146	545,663	30,084	190,871	12,578	10,435	62.90%	91.52%	74.56%
			20x	373,284	131,217	644,975	25,638	235,809	9,434	11,631	73.99%	93.73%	82.70%
			30x	395,198	109,303	692,424	25,769	259,975	9,135	12,848	78.33%	94.04%	85.47%
			40x	407,634	96,867	722,948	26,519	276,614	9,500	13,761	80.80%	94.06%	86.93%
			50x	415,558	88,943	743,228	26,836	288,220	9,813	14,125	82.37%	94.10%	87.85%
	HG004	510,519	10x	315,798	194,721	542,162	30,510	188,642	12,920	10,641	61.86%	91.37%	73.77%
			20x	374,443	136,076	645,439	25,775	235,058	9,317	11,954	73.35%	93.72%	82.29%
			30x	396,487	114,032	691,800	26,045	257,858	9,124	13,164	77.66%	94.00%	85.05%
			40x	408,827	101,692	721,084	26,821	273,287	9,464	14,232	80.08%	94.01%	86.49%
			50x	417,129	93,390	742,286	27,190	285,330	9,732	14,669	81.71%	94.05%	87.44%
	HG005	416,777	10x	301,753	115,024	536,421	26,229	202,086	11,486	8,150	72.40%	92.15%	81.09%
			20x	352,016	64,761	637,290	18,930	257,455	7,769	7,294	84.46%	95.02%	89.43%
			30x	368,605	48,172	685,577	16,279	290,810	6,541	6,736	88.44%	95.88%	92.01%
			40x	376,878	39,899	716,865	14,750	314,838	5,911	6,398	90.43%	96.33%	93.29%
			50x	381,528	35,249	736,786	13,681	330,861	5,581	6,088	91.54%	96.63%	94.02%
	HG006	428,237	10x	291,574	136,663	542,029	24,735	219,417	10,391	7,913	68.09%	92.33%	78.38%
			20x	338,852	89,386	641,810	19,255	274,958	6,844	8,238	79.13%	94.75%	86.24%
			30x	356,052	72,186	690,179	18,007	306,408	5,901	8,674	83.14%	95.31%	88.81%
			40x	364,895	63,343	720,816	17,861	327,886	5,857	9,021	85.21%	95.45%	90.04%
			50x	370,557	57,681	741,980	17,739	343,176	5,976	9,134	86.53%	95.55%	90.82%
	HG007	430,326	10x	290,345	139,981	542,676	25,071	220,852	10,698	7,832	67.47%	92.21%	77.92%
			20x	337,410	92,916	642,393	19,808	276,459	7,159	8,475	78.41%	94.59%	85.74%
			30x	354,379	75,947	689,777	18,736	307,015	6,258	9,204	82.35%	95.11%	88.27%
			40x	362,983	67,343	719,379	18,517	327,742	6,248	9,510	84.35%	95.27%	89.48%
			50x	361,777	68,549	733,353	28,926	332,296	7,639	10,945	84.07%	92.79%	88.21%

Supplementary table 2 *Clair3 baseline (HAC) performance*

Type	Sample	TRUTH.TOTAL	Depth	TRUTH.TP	TRUTH.FN	QUERY.TOTAL	QUERY.FP	QUERY.UNK	FP.gt	FP.al	METRIC.Recall	METRIC.Precision	METRIC.F1_Score
SNP	HG001	3,254,386	10x	3,051,760	202,626	3,825,515	46,054	727,428	31,562	2,705	93.77%	98.51%	96.09%
			20x	3,214,684	39,702	4,024,045	14,043	794,970	6,946	1,171	98.78%	99.57%	99.17%
			30x	3,233,756	20,630	4,076,642	9,576	832,967	4,915	610	99.37%	99.70%	99.54%
			40x	3,239,150	15,236	4,100,000	8,765	851,717	4,342	502	99.53%	99.73%	99.63%
			50x	3,241,071	13,315	4,118,035	7,874	868,754	4,150	441	99.59%	99.76%	99.67%
	HG003	3,327,495	10x	3,152,420	175,075	3,892,164	41,750	697,464	27,825	3,031	94.74%	98.69%	96.68%
			20x	3,288,252	39,243	4,072,741	14,959	768,889	6,880	1,741	98.82%	99.55%	99.18%
			30x	3,302,908	24,587	4,125,233	11,419	810,257	5,421	1,032	99.26%	99.66%	99.46%
			40x	3,306,474	21,021	4,145,060	9,844	828,087	4,989	728	99.37%	99.70%	99.54%
			50x	3,307,846	19,649	4,158,599	9,344	840,767	4,824	665	99.41%	99.72%	99.56%
	HG004	3,346,610	10x	3,155,219	191,391	3,892,149	41,714	694,634	30,149	2,929	94.28%	98.70%	96.44%
			20x	3,306,733	39,877	4,093,281	13,523	772,299	6,713	1,506	98.81%	99.59%	99.20%
			30x	3,323,749	22,861	4,147,109	9,730	812,945	5,071	983	99.32%	99.71%	99.51%
			40x	3,328,379	18,231	4,166,347	8,492	828,758	4,641	780	99.46%	99.75%	99.60%
			50x	3,329,999	16,611	4,188,795	8,023	850,066	4,433	781	99.50%	99.76%	99.63%
	HG005	3,275,631	10x	3,091,027	184,604	3,863,569	46,024	726,089	31,093	2,748	94.36%	98.53%	96.40%
			20x	3,237,317	38,314	4,044,217	13,915	792,508	7,338	1,218	98.83%	99.57%	99.20%
			30x	3,255,054	20,577	4,105,183	10,938	838,658	5,296	891	99.37%	99.67%	99.52%
			40x	3,259,643	15,988	4,121,060	9,829	851,038	4,909	664	99.51%	99.70%	99.61%
			50x	3,261,167	14,464	4,136,162	8,750	865,704	4,831	604	99.56%	99.73%	99.65%
	HG006	3,269,860	10x	3,094,067	175,793	3,837,217	43,916	698,931	30,689	2,381	94.62%	98.60%	96.57%
			20x	3,233,864	35,996	4,017,273	14,480	768,601	7,386	1,018	98.90%	99.55%	99.23%
			30x	3,249,729	20,131	4,066,130	10,543	805,549	5,178	591	99.38%	99.68%	99.53%
			40x	3,253,770	16,090	4,096,668	10,454	832,154	4,903	534	99.51%	99.68%	99.59%
			50x	3,255,118	14,742	4,109,476	9,479	844,594	4,676	450	99.55%	99.71%	99.63%
	HG007	3,284,462	10x	3,105,808	178,654	3,858,409	44,171	708,175	32,486	2,265	94.56%	98.60%	96.54%
			20x	3,244,677	39,785	4,034,654	13,544	776,110	7,720	943	98.79%	99.58%	99.18%
			30x	3,261,365	23,097	4,076,178	9,891	804,661	5,369	599	99.30%	99.70%	99.50%
			40x	3,265,430	19,032	4,099,790	8,517	825,572	4,877	473	99.42%	99.74%	99.58%
			50x	3,265,436	19,026	4,115,213	9,169	840,365	5,231	505	99.42%	99.72%	99.57%
INDEL	HG001	467,702	10x	208,617	259,085	313,686	8,201	95,064	4,334	1,342	44.60%	96.25%	60.96%
			20x	284,022	183,680	435,781	7,212	140,377	3,972	1,835	60.73%	97.56%	74.86%
			30x	309,962	157,740	482,710	7,148	159,893	4,125	2,170	66.27%	97.79%	79.00%
			40x	323,307	144,395	507,788	7,021	170,936	4,128	2,301	69.13%	97.92%	81.04%
			50x	329,985	137,717	520,063	6,696	176,470	4,007	2,237	70.55%	98.05%	82.06%
	HG003	504,501	10x	232,475	272,026	340,145	8,864	96,265	4,700	1,534	46.08%	96.37%	62.35%
			20x	307,241	197,260	458,435	7,460	138,109	4,126	2,048	60.90%	97.67%	75.02%
			30x	332,203	172,298	502,058	7,279	155,307	4,221	2,304	65.85%	97.90%	78.74%
			40x	344,684	159,817	524,714	7,312	164,512	4,317	2,494	68.32%	97.97%	80.50%
			50x	351,041	153,460	535,636	7,219	168,803	4,350	2,493	69.58%	98.03%	81.39%
	HG004	510,519	10x	229,250	281,269	334,233	9,204	93,441	4,872	1,570	44.91%	96.18%	61.22%
			20x	306,699	203,820	456,826	7,521	137,199	4,087	2,043	60.08%	97.65%	74.39%
			30x	332,299	178,220	501,053	7,189	154,416	4,044	2,354	65.09%	97.93%	78.20%
			40x	345,704	164,815	525,462	7,206	164,437	4,134	2,619	67.72%	98.00%	80.09%
			50x	352,657	157,862	537,838	7,090	169,535	4,101	2,612	69.08%	98.08%	81.06%
	HG005	416,777	10x	219,075	197,702	325,872	8,151	96,531	4,469	1,187	52.56%	96.45%	68.04%
			20x	290,561	126,216	441,969	7,008	139,822	4,101	1,575	69.72%	97.68%	81.36%
			30x	315,055	101,722	487,220	6,531	159,438	4,078	1,649	75.59%	98.01%	85.35%
			40x	326,927	89,850	510,423	6,504	170,061	4,225	1,700	78.44%	98.09%	87.17%
			50x	332,963	83,814	521,865	6,306	175,299	4,169	1,675	79.89%	98.18%	88.10%
	HG006	428,237	10x	217,979	210,258	337,112	7,976	109,004	4,246	1,269	50.90%	96.50%	66.65%
			20x	285,696	142,542	452,691	6,552	155,739	3,635	1,615	66.71%	97.79%	79.32%
			30x	307,749	120,489	496,132	6,045	176,066	3,454	1,827	71.86%	98.11%	82.96%
			40x	318,369	109,869	519,132	6,001	187,664	3,580	1,840	74.34%	98.19%	84.62%
			50x	323,650	104,588	530,099	5,844	193,162	3,518	1,840	75.58%	98.27%	85.44%
	HG007	430,326	10x	217,855	212,471	338,964	8,187	110,688	4,245	1,286	50.63%	96.41%	66.39%
			20x	285,101	145,225	454,794	6,703	158,164	3,668	1,725	66.25%	97.74%	78.97%
			30x	306,633	123,693	496,599	6,029	177,631	3,514	1,862	71.26%	98.11%	82.55%
			40x	317,082	113,244	519,539	6,137	189,264	3,634	2,050	73.68%	98.14%	84.17%
			50x	317,105	113,221	525,761	8,548	192,765	4,540	2,558	73.69%	97.43%	83.91%

Supplementary table 3 *Clair3 v2 (SUP) performance*

Type	Sample	TRUTH.TOTAL	Depth	TRUTH.TP	TRUTH.FN	QUERY.TOTAL	QUERY.FP	QUERY.UNK	FP.gt	FP.al	METRIC.Recall	METRIC.Precision	METRIC.F1_Score
SNP	HG001	3,254,386	10x	3,167,968	86,418	4,051,017	33,142	849,191	20,166	2,055	97.34%	98.96%	98.15%
			20x	3,241,958	12,428	4,210,748	7,566	960,129	1,385	907	99.62%	99.77%	99.69%
			30x	3,247,237	7,149	4,276,544	5,521	1,022,574	787	605	99.78%	99.83%	99.81%
			40x	3,249,447	4,939	4,326,246	5,348	1,070,208	777	547	99.85%	99.84%	99.84%
			50x	3,250,704	3,682	4,365,929	4,245	1,109,676	724	441	99.89%	99.87%	99.88%
	HG003	3,327,495	10x	3,246,353	81,142	4,104,739	32,993	824,408	19,429	2,817	97.56%	98.99%	98.27%
			20x	3,308,951	18,544	4,259,071	8,327	940,347	1,708	1,333	99.44%	99.75%	99.60%
			30x	3,313,597	13,898	4,332,755	6,003	1,011,545	1,146	851	99.58%	99.82%	99.70%
			40x	3,315,994	11,501	4,383,838	5,324	1,060,846	1,132	757	99.65%	99.84%	99.75%
			50x	3,317,620	9,875	4,428,419	4,947	1,104,142	1,155	712	99.70%	99.85%	99.78%
	HG004	3,346,610	10x	3,257,583	89,027	4,110,636	31,737	820,249	20,981	2,387	97.34%	99.04%	98.18%
			20x	3,328,447	18,163	4,270,054	7,320	932,776	1,723	1,175	99.46%	99.78%	99.62%
			30x	3,334,059	12,551	4,346,468	4,987	1,005,763	1,108	840	99.63%	99.85%	99.74%
			40x	3,337,198	9,412	4,400,495	4,166	1,057,418	1,101	744	99.72%	99.88%	99.80%
			50x	3,338,935	7,675	4,443,723	3,588	1,099,460	1,105	720	99.77%	99.89%	99.83%
	HG005	3,275,631	10x	3,198,969	76,662	4,082,361	31,612	850,900	18,414	2,160	97.66%	99.02%	98.34%
			20x	3,261,567	14,064	4,227,522	7,201	957,500	1,587	953	99.57%	99.78%	99.68%
			30x	3,267,179	8,452	4,306,302	5,150	1,032,637	825	704	99.74%	99.84%	99.79%
			40x	3,269,297	6,334	4,363,187	4,667	1,087,806	804	618	99.81%	99.86%	99.83%
			50x	3,270,410	5,221	4,402,352	4,001	1,126,493	821	574	99.84%	99.88%	99.86%
	HG006	3,269,860	10x	3,192,027	77,833	4,042,617	34,285	815,771	19,564	1,934	97.62%	98.94%	98.27%
			20x	3,254,894	14,966	4,184,207	7,771	920,762	1,756	781	99.54%	99.76%	99.65%
			30x	3,259,919	9,941	4,266,016	5,388	999,881	999	599	99.70%	99.84%	99.77%
			40x	3,262,368	7,492	4,323,625	4,976	1,055,429	995	567	99.77%	99.85%	99.81%
			50x	3,263,603	6,257	4,368,613	4,629	1,099,507	1,005	502	99.81%	99.86%	99.83%
	HG007	3,284,462	10x	3,203,440	81,022	4,056,765	31,987	820,863	20,780	1,812	97.53%	99.01%	98.27%
			20x	3,266,102	18,360	4,192,091	6,919	918,369	1,812	834	99.44%	99.79%	99.61%
			30x	3,271,723	12,739	4,261,007	4,796	983,702	984	591	99.61%	99.85%	99.73%
			40x	3,274,449	10,013	4,317,706	4,411	1,038,022	953	501	99.70%	99.87%	99.78%
			50x	3,276,269	8,193	4,363,558	3,957	1,082,500	970	474	99.75%	99.88%	99.81%
INDEL	HG001	467,702	10x	312,437	155,265	559,207	25,088	215,536	12,377	7,792	66.80%	92.70%	77.65%
			20x	369,307	98,395	673,181	23,668	271,040	10,240	9,552	78.96%	94.11%	85.88%
			30x	388,730	78,972	719,059	24,023	295,876	10,103	10,682	83.11%	94.32%	88.37%
			40x	399,549	68,153	746,592	23,703	312,104	9,845	11,020	85.43%	94.54%	89.76%
			50x	406,365	61,337	764,322	23,550	322,748	9,760	11,276	86.89%	94.67%	90.61%
	HG003	504,501	10x	336,949	167,552	581,511	27,643	208,952	13,688	8,676	66.79%	92.58%	77.60%
			20x	393,182	111,319	686,209	25,809	256,139	11,359	10,643	77.93%	94.00%	85.22%
			30x	413,546	90,955	729,390	25,848	277,585	10,992	11,782	81.97%	94.28%	87.70%
			40x	424,511	79,990	754,718	25,639	291,354	10,786	12,209	84.14%	94.47%	89.01%
			50x	431,789	72,712	771,567	25,677	300,515	10,798	12,420	85.59%	94.55%	89.85%
	HG004	510,519	10x	335,872	174,647	577,438	27,842	205,900	13,929	8,802	65.79%	92.51%	76.89%
			20x	393,885	116,634	685,098	25,865	254,201	11,133	10,889	77.15%	94.00%	84.75%
			30x	414,336	96,183	726,328	25,784	273,730	10,860	12,025	81.16%	94.30%	87.24%
			40x	426,064	84,455	752,199	25,627	287,351	10,700	12,577	83.46%	94.49%	88.63%
			50x	433,887	76,632	769,851	25,551	296,823	10,539	12,874	84.99%	94.60%	89.54%
	HG005	416,777	10x	318,752	98,025	572,191	22,606	223,966	12,069	6,153	76.48%	93.51%	84.14%
			20x	366,474	50,303	679,817	16,610	287,161	8,423	5,543	87.93%	95.77%	91.68%
			30x	380,766	36,011	725,133	13,644	320,165	6,872	4,948	91.36%	96.63%	93.92%
			40x	387,534	29,243	751,004	12,011	340,448	6,067	4,540	92.98%	97.07%	94.99%
			50x	391,699	25,078	767,723	11,023	353,719	5,552	4,253	93.98%	97.34%	95.63%
	HG006	428,237	10x	306,570	121,668	576,385	22,399	240,721	11,029	6,378	71.59%	93.33%	81.03%
			20x	352,774	75,464	682,143	18,966	301,153	7,991	7,342	82.38%	95.02%	88.25%
			30x	368,436	59,802	728,163	18,120	331,388	7,234	7,940	86.04%	95.43%	90.49%
			40x	376,103	52,135	753,390	17,246	349,297	6,766	7,948	87.83%	95.73%	91.61%
			50x	381,562	46,676	770,120	16,726	360,816	6,488	7,938	89.10%	95.91%	92.38%
	HG007	430,326	10x	306,268	124,058	578,077	22,589	242,380	11,025	6,592	71.17%	93.27%	80.74%
			20x	351,768	78,558	682,171	19,002	302,055	7,861	7,631	81.74%	95.00%	87.88%
			30x	366,308	64,018	722,554	17,879	328,174	7,224	8,017	85.12%	95.47%	90.00%
			40x	374,781	55,545	749,539	17,382	346,643	6,932	8,090	87.09%	95.69%	91.19%
			50x	380,556	49,770	768,128	17,239	359,342	6,775	8,258	88.43%	95.78%	91.96%

Supplementary table 4 *Clair3 baseline (SUP) performance*

Type	Sample	TRUTH.TOTAL	Depth	TRUTH.TP	TRUTH.FN	QUERY.TOTAL	QUERY.FP	QUERY.UNK	FP.gt	FP.al	METRIC.Recall	METRIC.Precision	METRIC.F1_Score
SNP	HG001	3,254,386	10x	3,156,650	97,736	3,999,484	35,078	807,238	21,839	1,608	97.00%	98.90%	97.94%
			20x	3,233,130	21,256	4,170,266	11,154	925,175	4,566	699	99.35%	99.66%	99.50%
			30x	3,239,627	14,759	4,233,949	8,407	985,034	3,534	474	99.55%	99.74%	99.64%
			40x	3,242,817	11,569	4,275,352	7,975	1,023,634	3,319	429	99.64%	99.75%	99.70%
			50x	3,245,828	8,558	4,315,725	5,993	1,062,955	2,121	366	99.74%	99.82%	99.78%
	HG003	3,327,495	10x	3,232,006	95,489	4,042,699	34,443	775,444	20,843	2,270	97.13%	98.95%	98.03%
			20x	3,297,116	30,379	4,203,264	11,487	893,507	5,037	1,074	99.09%	99.65%	99.37%
			30x	3,304,157	23,338	4,277,583	9,014	963,123	3,725	737	99.30%	99.73%	99.51%
			40x	3,308,906	18,589	4,319,473	7,821	1,001,360	3,276	585	99.44%	99.76%	99.60%
			50x	3,312,391	15,104	4,357,381	6,729	1,036,895	2,519	620	99.55%	99.80%	99.67%
	HG004	3,346,610	10x	3,241,544	105,066	4,054,391	34,063	777,929	23,390	1,847	96.86%	98.96%	97.90%
			20x	3,315,381	31,229	4,227,149	11,193	899,346	5,355	909	99.07%	99.66%	99.36%
			30x	3,323,344	23,266	4,296,086	8,289	963,120	4,152	697	99.30%	99.75%	99.53%
			40x	3,329,047	17,563	4,346,758	6,698	1,009,651	3,559	617	99.48%	99.80%	99.64%
			50x	3,332,701	13,909	4,391,176	5,664	1,051,373	2,813	596	99.58%	99.83%	99.71%
	HG005	3,275,631	10x	3,187,921	87,710	4,033,123	33,268	811,199	19,469	1,699	97.32%	98.97%	98.14%
			20x	3,251,995	23,636	4,180,266	10,570	916,666	4,951	707	99.28%	99.68%	99.48%
			30x	3,258,477	17,154	4,252,324	8,750	983,986	3,977	619	99.48%	99.73%	99.60%
			40x	3,262,329	13,302	4,305,768	7,620	1,034,676	3,386	540	99.59%	99.77%	99.68%
			50x	3,265,054	10,577	4,348,935	6,572	1,076,176	2,691	494	99.68%	99.80%	99.74%
	HG006	3,269,860	10x	3,179,957	89,903	3,992,292	35,280	776,637	20,305	1,651	97.25%	98.90%	98.07%
			20x	3,243,847	26,013	4,137,678	11,556	881,696	5,079	665	99.20%	99.65%	99.42%
			30x	3,250,032	19,828	4,219,484	9,030	959,773	4,149	512	99.39%	99.72%	99.56%
			40x	3,254,598	15,262	4,274,385	8,683	1,010,428	3,601	529	99.53%	99.73%	99.63%
			50x	3,257,855	12,005	4,311,895	7,026	1,046,326	2,766	450	99.63%	99.78%	99.71%
	HG007	3,284,462	10x	3,190,577	93,885	4,004,241	32,935	780,391	21,172	1,437	97.14%	98.98%	98.05%
			20x	3,254,713	29,749	4,147,757	10,203	882,344	4,697	652	99.09%	99.69%	99.39%
			30x	3,261,346	23,116	4,207,326	8,328	937,092	4,070	493	99.30%	99.75%	99.52%
			40x	3,266,605	17,857	4,269,809	7,211	995,391	3,436	435	99.46%	99.78%	99.62%
			50x	3,270,349	14,113	4,312,548	5,995	1,035,585	2,643	408	99.57%	99.82%	99.69%
INDEL	HG001	467,702	10x	302,763	164,939	495,245	15,430	172,586	6,727	3,912	64.73%	95.22%	77.07%
			20x	358,114	109,588	597,014	11,629	220,389	4,645	4,156	76.57%	96.91%	85.55%
			30x	376,752	90,950	637,358	10,983	241,611	4,158	4,606	80.55%	97.22%	88.11%
			40x	387,302	80,400	660,953	10,572	254,347	3,943	4,843	82.81%	97.40%	89.51%
			50x	394,396	73,306	677,505	10,500	263,519	3,798	5,059	84.33%	97.46%	90.42%
	HG003	504,501	10x	324,992	179,509	514,406	16,829	166,776	7,256	4,557	64.42%	95.16%	76.83%
			20x	379,958	124,543	607,678	12,595	206,689	5,028	4,764	75.31%	96.86%	84.74%
			30x	399,586	104,915	647,007	12,088	225,575	4,709	5,272	79.20%	97.13%	87.26%
			40x	410,591	93,910	669,463	11,771	236,732	4,455	5,605	81.39%	97.28%	88.63%
			50x	418,095	86,406	685,023	11,954	244,231	4,448	5,896	82.87%	97.29%	89.50%
	HG004	510,519	10x	323,802	186,717	511,068	17,094	164,506	7,567	4,515	63.43%	95.07%	76.09%
			20x	380,421	130,098	607,883	12,840	206,103	5,076	4,794	74.52%	96.80%	84.21%
			30x	399,860	110,659	645,048	12,281	223,166	4,604	5,473	78.32%	97.09%	86.70%
			40x	411,521	98,998	668,949	12,232	234,684	4,513	5,886	80.61%	97.18%	88.12%
			50x	419,354	91,165	686,040	12,396	243,405	4,414	6,250	82.14%	97.20%	89.04%
	HG005	416,777	10x	309,324	107,453	506,588	14,134	178,100	6,247	3,306	74.22%	95.70%	83.60%
			20x	357,966	58,811	601,594	8,804	227,400	3,706	2,823	85.89%	97.65%	91.39%
			30x	372,667	44,110	641,528	7,200	253,183	2,896	2,686	89.42%	98.15%	93.58%
			40x	379,413	37,364	663,551	6,166	268,990	2,467	2,471	91.04%	98.44%	94.59%
			50x	383,528	33,249	678,400	5,757	279,900	2,265	2,434	92.02%	98.56%	95.18%
	HG006	428,237	10x	297,865	130,373	509,448	13,932	192,606	5,789	3,414	69.56%	95.60%	80.53%
			20x	343,746	84,492	603,389	9,359	243,001	3,493	3,344	80.27%	97.40%	88.01%
			30x	358,883	69,355	642,506	8,224	267,193	2,882	3,520	83.80%	97.81%	90.27%
			40x	366,912	61,326	664,307	7,675	281,106	2,611	3,634	85.68%	98.00%	91.43%
			50x	372,738	55,500	679,886	7,461	290,817	2,499	3,734	87.04%	98.08%	92.23%
	HG007	430,326	10x	297,049	133,277	510,791	14,215	194,492	5,890	3,603	69.03%	95.51%	80.14%
			20x	342,616	87,710	604,147	9,700	244,446	3,514	3,608	79.62%	97.30%	87.58%
			30x	356,825	73,501	639,556	8,415	266,078	3,054	3,607	82.92%	97.75%	89.72%
			40x	365,421	64,905	663,276	8,223	280,928	2,848	3,887	84.92%	97.85%	90.93%
			50x	371,478	58,848	679,978	8,120	291,425	2,702	4,053	86.32%	97.91%	91.75%

Supplementary table 5 *Clair3 (HAC) indel performance comparison stratified by length*

		F1 score		Recall		Precision	
Depth	Category	With dwelling time	Without dwelling time	With dwelling time	Without dwelling time	With dwelling time	Without dwelling time
10x	SNP	97.78%	96.40%	96.80%	94.36%	98.78%	98.53%
	Del 1-5bp	80.34%	67.12%	70.73%	51.18%	92.98%	97.47%
	Del 6-15bp	85.47%	68.81%	80.95%	53.67%	90.53%	95.86%
	Del 16+bp	88.84%	70.36%	83.14%	55.02%	95.39%	97.56%
	Ins 1-5bp	79.86%	68.28%	70.62%	53.07%	91.88%	95.72%
	Ins 6-15bp	82.04%	54.88%	79.18%	39.09%	85.10%	92.08%
	Ins 16+bp	87.46%	63.46%	83.79%	47.65%	91.47%	94.95%
	All INDELs	81.09%	68.04%	72.40%	52.56%	92.15%	96.45%
20x	SNP	99.51%	99.20%	99.28%	98.83%	99.74%	99.57%
	Del 1-5bp	88.99%	80.92%	83.31%	68.63%	95.50%	98.58%
	Del 6-15bp	93.51%	84.98%	92.02%	75.66%	95.05%	96.92%
	Del 16+bp	95.63%	86.60%	93.67%	77.61%	97.67%	97.93%
	Ins 1-5bp	88.32%	80.34%	82.88%	68.46%	94.53%	97.21%
	Ins 6-15bp	91.46%	74.72%	91.25%	62.70%	91.67%	92.44%
	Ins 16+bp	94.52%	83.05%	93.22%	72.86%	95.85%	96.56%
	All INDELs	89.43%	81.36%	84.46%	69.72%	95.02%	97.68%
30x	SNP	99.67%	99.52%	99.53%	99.37%	99.81%	99.67%
	Del 1-5bp	91.64%	85.43%	87.49%	75.21%	96.20%	98.86%
	Del 6-15bp	95.74%	89.87%	94.91%	83.40%	96.59%	97.42%
	Del 16+bp	97.23%	90.36%	96.11%	83.88%	98.39%	97.92%
	Ins 1-5bp	91.10%	83.80%	87.21%	73.42%	95.37%	97.61%
	Ins 6-15bp	94.23%	80.99%	94.40%	72.02%	94.06%	92.50%
	Ins 16+bp	96.28%	87.61%	95.55%	80.00%	97.03%	96.81%
	All INDELs	92.01%	85.35%	88.44%	75.59%	95.88%	98.01%
40x	SNP	99.74%	99.61%	99.65%	99.51%	99.83%	99.70%
	Del 1-5bp	92.97%	87.53%	89.63%	78.49%	96.56%	98.92%
	Del 6-15bp	96.78%	91.96%	96.21%	86.80%	97.35%	97.78%
	Del 16+bp	97.89%	91.29%	97.15%	85.84%	98.64%	97.49%
	Ins 1-5bp	92.46%	85.41%	89.33%	75.86%	95.82%	97.70%
	Ins 6-15bp	95.57%	83.52%	95.76%	76.36%	95.38%	92.16%
	Ins 16+bp	97.33%	89.46%	96.83%	83.05%	97.83%	96.94%
	All INDELs	93.29%	87.17%	90.43%	78.44%	96.33%	98.09%
50x	SNP	99.81%	99.65%	99.75%	99.56%	99.86%	99.73%
	Del 1-5bp	93.80%	88.66%	90.95%	80.28%	96.83%	99.00%
	Del 6-15bp	97.34%	92.82%	96.81%	88.17%	97.88%	98.00%
	Del 16+bp	98.09%	91.88%	97.39%	86.64%	98.80%	97.80%
	Ins 1-5bp	93.21%	86.21%	90.47%	77.07%	96.12%	97.81%
	Ins 6-15bp	96.04%	84.39%	96.22%	78.00%	95.86%	91.92%
	Ins 16+bp	97.54%	89.99%	97.01%	83.92%	98.06%	97.02%
	All INDELs	94.02%	88.10%	91.54%	79.89%	96.63%	98.18%

Supplementary table 6 *Clair3 (SUP) indel performance comparison stratified by length*

		F1 score		Recall		Precision	
**Depth**	**Category**	**With dwelling time**	**Without dwelling time**	**With dwelling time**	**Without dwelling time**	**With dwelling time**	**Without dwelling time**
	SNP	98.34%	98.14%	97.66%	97.32%	99.02%	98.97%
	Del 1-5bp	83.83%	82.70%	75.73%	72.29%	93.86%	96.61%
	Del 6-15bp	87.67%	81.46%	84.37%	70.47%	91.24%	96.52%
	Del 16+bp	92.22%	83.22%	88.52%	72.45%	96.25%	97.74%
	Ins 1-5bp	82.83%	83.59%	74.05%	74.80%	93.98%	94.73%
	Ins 6-15bp	82.81%	81.38%	79.52%	71.63%	86.38%	94.20%
	Ins 16+bp	89.99%	88.19%	86.82%	81.78%	93.40%	95.71%
10x	All INDELs	84.14%	83.60%	76.48%	74.22%	93.51%	95.70%
	SNP	99.68%	99.48%	99.57%	99.28%	99.78%	99.68%
	Del 1-5bp	91.48%	90.78%	87.57%	84.40%	95.75%	98.21%
	Del 6-15bp	95.08%	91.70%	94.30%	85.74%	95.87%	98.56%
	Del 16+bp	97.40%	94.48%	96.65%	90.75%	98.16%	98.53%
	Ins 1-5bp	90.68%	91.06%	85.98%	85.84%	95.93%	96.96%
	Ins 6-15bp	91.76%	90.76%	91.52%	85.01%	92.00%	97.34%
	Ins 16+bp	96.22%	94.04%	95.55%	90.86%	96.91%	97.46%
20x	All INDELs	91.68%	91.39%	87.93%	85.89%	95.77%	97.65%
	SNP	99.79%	99.60%	99.74%	99.48%	99.84%	99.73%
	Del 1-5bp	93.77%	93.16%	91.25%	88.28%	96.43%	98.61%
	Del 6-15bp	96.98%	94.29%	96.57%	89.95%	97.39%	99.06%
	Del 16+bp	98.16%	96.24%	97.75%	93.91%	98.58%	98.69%
	Ins 1-5bp	93.08%	93.22%	89.67%	89.28%	96.77%	97.53%
	Ins 6-15bp	94.42%	93.20%	94.39%	88.65%	94.45%	98.24%
	Ins 16+bp	97.65%	95.08%	97.28%	92.38%	98.03%	97.94%
30x	All INDELs	93.92%	93.58%	91.36%	89.42%	96.63%	98.15%
	SNP	99.83%	99.68%	99.81%	99.59%	99.86%	99.77%
	Del 1-5bp	94.78%	94.28%	92.87%	90.15%	96.77%	98.82%
	Del 6-15bp	97.87%	95.19%	97.50%	91.46%	98.25%	99.23%
	Del 16+bp	98.43%	96.30%	98.27%	94.04%	98.59%	98.67%
	Ins 1-5bp	94.32%	94.27%	91.58%	90.88%	97.22%	97.93%
	Ins 6-15bp	95.65%	94.25%	95.67%	90.30%	95.63%	98.56%
	Ins 16+bp	98.09%	95.55%	97.67%	92.96%	98.50%	98.30%
40x	All INDELs	94.99%	94.59%	92.98%	91.04%	97.07%	98.44%
	SNP	99.86%	99.74%	99.84%	99.68%	99.88%	99.80%
	Del 1-5bp	95.46%	94.94%	93.94%	91.27%	97.03%	98.92%
	Del 6-15bp	98.38%	95.55%	98.06%	91.95%	98.70%	99.45%
	Del 16+bp	98.64%	96.24%	98.46%	93.84%	98.83%	98.77%
	Ins 1-5bp	94.99%	94.87%	92.67%	91.90%	97.43%	98.05%
	Ins 6-15bp	96.30%	94.79%	96.33%	91.16%	96.28%	98.72%
	Ins 16+bp	98.28%	95.81%	97.98%	93.39%	98.59%	98.35%
50x	All INDELs	95.63%	95.18%	93.98%	92.02%	97.34%	98.56%

***Supplementary table 7** Clair3 v2 performance in complex regions (HG005)*

Variant type	Enable dwellign time?	Stratification type	Region	TP	FP	FN	Precision	Recall	F1
			Homopol_4-6bp	112,166	2,970	8,143	97.46%	93.23%	95.30%
			Homopol_7-11bp	79,649	5,123	13,238	93.99%	85.75%	89.68%
			Homopol_ge12bp	14,356	6,148	28,730	70.39%	33.32%	45.23%
			Imp_Homopol_ge11bp	40,806	8,643	35,721	82.77%	53.32%	64.86%
			TR_le50bp	57,080	3,084	3,496	95.35%	94.23%	94.79%
			TR51-200bp	25,693	1,348	1,814	95.46%	93.41%	94.42%
			TR201-10kbp	3,981	49	100	98.82%	97.55%	98.18%
			TR_ge101bp	11,674	299	516	97.63%	95.77%	96.69%
		LowComplexity	TR_and_Homopol	186,577	15,712	46,968	92.59%	79.89%	85.77%
			SegDups	9,962	359	399	96.57%	96.15%	96.36%
		SegmentalDuplications	SegDups_gt10kb	8,472	332	358	96.28%	95.95%	96.11%
		Mappability	LowMap	9,500	217	299	97.77%	96.95%	97.36%
			L1H	196	1	3	99.49%	98.49%	98.99%
		OtherDifficult	MHC	1,552	72	146	95.87%	91.40%	93.58%
	Yes	FunctionalRegions	CDS	352	5	5	98.63%	98.60%	98.61%
			Homopol_4-6bp	100,733	1,433	19,573	98.61%	83.73%	90.56%
			Homopol_7-11bp	58,468	1,727	34,418	97.13%	62.95%	76.39%
			Homopol_ge12bp	3,374	763	39,712	81.49%	7.83%	14.29%
			Imp_Homopol_ge11bp	20,628	1,520	55,899	93.17%	26.96%	41.81%
			TR_le50bp	45,919	2,357	14,657	95.51%	75.80%	84.52%
			TR51-200bp	20,707	824	6,800	96.46%	75.28%	84.56%
			TR201-10kbp	3,632	57	449	98.49%	89.00%	93.51%
			TR_ge101bp	10,063	232	2,127	97.84%	82.55%	89.55%
		LowComplexity	TR_and_Homopol	137,413	5,809	96,132	96.11%	58.84%	72.99%
			SegDups	9,230	279	1,131	97.10%	89.08%	92.92%
		SegmentalDuplications	SegDups_gt10kb	7,852	259	978	96.84%	88.92%	92.72%
		Mappability	LowMap	8,990	188	809	97.95%	91.74%	94.75%
			L1H	186	1	13	99.47%	93.47%	96.37%
		OtherDifficult	MHC	1,389	57	309	96.30%	81.80%	88.46%
Indel	No	FunctionalRegions	CDS	324	4	33	98.78%	90.76%	94.60%
			Homopol_4-6bp	812,358	1,623	3,729	99.80%	99.54%	99.67%
			Homopol_7-11bp	62,894	547	794	99.14%	98.75%	98.95%
			Homopol_ge12bp	5,064	434	751	92.27%	87.09%	89.60%
			Imp_Homopol_ge11bp	27,187	650	1,091	97.68%	96.14%	96.91%
			TR_le50bp	30,787	131	283	99.58%	99.09%	99.33%
			TR51-200bp	24,782	124	254	99.51%	98.99%	99.25%
			TR201-10kbp	10,212	36	39	99.65%	99.62%	99.63%
			TR_ge101bp	19,702	66	136	99.67%	99.31%	99.49%
		LowComplexity	TR_and_Homopol	146,118	1,224	2,118	99.18%	98.57%	98.87%
			SegDups	121,534	3,319	2,581	97.34%	97.92%	97.63%
		SegmentalDuplications	SegDups_gt10kb	104,972	3,301	2,535	96.95%	97.64%	97.30%
		Mappability	LowMap	190,184	2,416	2,778	98.75%	98.56%	98.65%
			L1H	5,574	5	29	99.83%	99.48%	99.70%
		OtherDifficult	MHC	18,685	43	200	99.77%	98.94%	99.35%
	Yes	FunctionalRegions	CDS	20,536	35	129	99.83%	99.38%	99.60%
			Homopol_4-6bp	809,263	3,268	6,824	99.60%	99.16%	99.38%
			Homopol_7-11bp	59,794	1,760	3,894	97.15%	93.89%	95.49%
			Homopol_ge12bp	3,856	875	1,959	81.69%	66.31%	73.20%
			Imp_Homopol_ge11bp	24,874	1,498	3,404	94.34%	87.96%	91.04%
			TR_le50bp	29,968	447	1,102	98.53%	96.45%	97.48%
			TR51-200bp	24,251	360	785	98.54%	96.86%	97.70%
			TR201-10kbp	10,165	68	86	99.33%	99.16%	99.25%
			TR_ge101bp	19,493	178	345	99.10%	98.26%	98.68%
		LowComplexity	TR_and_Homopol	140,361	3,477	7,875	97.59%	94.69%	96.12%
			SegDups	121,068	4,053	3,047	96.76%	97.55%	97.15%
		SegmentalDuplications	SegDups_gt10kb	104,523	3,996	2,984	96.32%	97.22%	96.77%
		Mappability	LowMap	189,805	2,904	3,157	98.49%	98.36%	98.43%
			L1H	5,573	8	30	99.86%	99.46%	99.66%
		OtherDifficult	MHC	18,530	94	355	99.49%	98.12%	98.80%
SNP	No	FunctionalRegions	CDS	20,575	61	90	99.70%	99.56%	99.63%

Supplementary Table 8 *Clair3 v2 performance in complex regions (HG001)*

Variant type	Enable dwellign time?	Stratification type	Region	TP	FP	FN	Precision	Recall	F1
Indel	Yes	LowComplexity	Homopol_4-6bp	111,116	3,913	14,950	96.64%	88.14%	92.20%
			Homopol_7-11bp	82,568	5,910	16,443	93.36%	83.39%	88.09%
			Homopol_ge12bp	21,954	13,211	73,730	62.88%	22.94%	33.62%
			Imp_Homopol_ge11bp	49,687	16,253	82,399	75.64%	37.62%	50.25%
			TR_le50bp	54,023	3,074	3,497	95.09%	93.92%	94.50%
			TR51-200bp	25,400	1,483	2,120	94.97%	92.30%	93.61%
			TR201-10kbp	4,141	61	114	98.59%	97.32%	97.95%
			TR_ge101bp	12,248	403	649	96.98%	94.97%	95.96%
			TR_and_Homopol	193,172	23,557	94,343	89.58%	67.19%	76.78%
		SegmentalDuplications	SegDups	8,850	380	290	95.94%	96.83%	96.38%
			SegDups_gt10kb	7,541	364	259	95.47%	96.68%	96.07%
		Mappability	LowMap	8,655	176	218	98.02%	97.54%	97.78%
		OtherDifficult	L1H	196	2	2	98.99%	98.99%	98.99%
			MHC	1,693	89	139	95.26%	92.41%	93.82%
		FunctionalRegions	CDS	351	10	5	97.22%	98.60%	97.90%
	No	LowComplexity	Homopol_4-6bp	98,436	1,500	27,623	98.51%	78.09%	87.12%
			Homopol_7-11bp	59,785	1,846	39,225	97.01%	60.38%	74.43%
			Homopol_ge12bp	4,191	1,302	91,493	76.21%	4.38%	8.28%
			Imp_Homopol_ge11bp	21,883	2,132	110,203	91.14%	16.57%	28.04%
			TR_le50bp	43,066	2,265	14,454	95.39%	74.87%	83.90%
			TR51-200bp	19,977	878	7,543	96.10%	72.59%	82.71%
			TR201-10kbp	3,762	65	493	98.34%	88.41%	93.12%
			TR_ge101bp	10,345	310	2,552	97.21%	80.21%	87.90%
			TR_and_Homopol	135,682	6,398	151,833	95.67%	47.19%	63.21%
		SegmentalDuplications	SegDups	8,124	333	1,016	96.11%	88.88%	92.36%
			SegDups_gt10kb	6,935	321	865	95.63%	88.91%	92.15%
		Mappability	LowMap	8,223	160	650	98.10%	92.67%	95.31%
		OtherDifficult	L1H	189	2	9	98.95%	95.45%	97.17%
			MHC	1,497	51	335	96.86%	81.71%	88.64%
		FunctionalRegions	CDS	321	4	35	98.76%	90.17%	94.27%
SNP	Yes	LowComplexity	Homopol_4-6bp	804,723	1,619	3,418	99.80%	99.58%	99.69%
			Homopol_7-11bp	62,327	623	891	99.02%	98.59%	98.80%
			Homopol_ge12bp	8,843	646	1,665	93.29%	84.15%	88.49%
			Imp_Homopol_ge11bp	31,932	942	2,087	97.16%	93.87%	95.48%
			TR_le50bp	30,313	122	280	99.60%	99.08%	99.34%
			TR51-200bp	25,580	147	298	99.43%	98.85%	99.14%
			TR201-10kbp	10,353	24	63	99.77%	99.40%	99.58%
			TR_ge101bp	20,292	71	167	99.65%	99.18%	99.42%
			TR_and_Homopol	149,819	1,517	3,159	99.00%	97.94%	98.47%
		SegmentalDuplications	SegDups	110,192	3,623	614	96.82%	99.45%	98.11%
			SegDups_gt10kb	94,074	3,594	566	96.32%	99.40%	97.84%
		Mappability	LowMap	174,871	1,931	851	98.91%	99.52%	99.21%
		OtherDifficult	L1H	5,454	7	28	99.87%	99.49%	99.68%
			MHC	20,283	37	167	99.82%	99.18%	99.50%
		FunctionalRegions	CDS	20,098	37	95	99.82%	99.53%	99.67%
	No	LowComplexity	Homopol_4-6bp	801,460	2,877	6,676	99.64%	99.17%	99.41%
			Homopol_7-11bp	59,240	1,662	3,979	97.28%	93.71%	95.46%
			Homopol_ge12bp	7,002	1,033	3,506	87.25%	66.63%	75.56%
			Imp_Homopol_ge11bp	28,847	1,673	5,172	94.54%	84.80%	89.40%
			TR_le50bp	29,577	328	1,016	98.90%	96.68%	97.78%
			TR51-200bp	25,027	359	851	98.59%	96.71%	97.64%
			TR201-10kbp	10,317	42	99	99.59%	99.05%	99.32%
			TR_ge101bp	20,073	156	386	99.23%	98.11%	98.67%
			TR_and_Homopol	143,504	3,380	9,474	97.70%	93.81%	95.72%
		SegmentalDuplications	SegDups	110,165	3,557	641	96.87%	99.42%	98.13%
			SegDups_gt10kb	94,048	3,521	592	96.39%	99.37%	97.86%
		Mappability	LowMap	174,883	1,935	839	98.91%	99.52%	99.21%
		OtherDifficult	L1H	5,461	14	21	99.74%	99.62%	99.68%
			MHC	20,131	85	319	99.58%	98.44%	99.01%
		FunctionalRegions	CDS	20,111	54	82	99.73%	99.59%	99.66%

Supplementary table 9 *Different variant caller comparison*

Varaint type	Depth	Variant caller	Sample	Region	TP	FP	FN	Precision	Recall	F1 score
		Clair3 V2	HG003	chr20	68,753	822	1,413	98.82%	97.99%	98.40%
		Dorado Variant	HG003	chr20	65,266	3,306	4,900	95.10%	93.02%	94.05%
		DeepVariant	HG003	chr20	67,233	656	2,933	99.03%	95.82%	97.40%
	10x	LongcallD	HG003	chr20	64,198	2,069	5,968	96.88%	91.49%	94.11%
		Clair3 V2	HG003	chr20	69,875	154	291	99.78%	99.59%	99.68%
		Dorado Variant	HG003	chr20	69,879	216	287	99.69%	99.59%	99.64%
		DeepVariant	HG003	chr20	69,994	137	172	99.80%	99.75%	99.78%
	20x	LongcallD	HG003	chr20	69,589	448	577	99.36%	99.18%	99.27%
		Clair3 V2	HG003	chr20	69,981	133	185	99.81%	99.74%	99.77%
		Dorado Variant	HG003	chr20	70,015	104	151	99.85%	99.78%	99.82%
		DeepVariant	HG003	chr20	70,070	129	96	99.82%	99.86%	99.84%
	30x	LongcallD	HG003	chr20	69,916	281	250	99.60%	99.64%	99.62%
		Clair3 V2	HG003	chr20	69,990	128	176	99.82%	99.75%	99.78%
		Dorado Variant	HG003	chr20	70,038	121	128	99.82%	99.82%	99.82%
		DeepVariant	HG003	chr20	70,075	123	91	99.82%	99.87%	99.85%
	40x	LongcallD	HG003	chr20	69,973	246	193	99.65%	99.72%	99.69%
		Clair3 V2	HG003	chr20	70,025	121	141	99.83%	99.80%	99.81%
		Dorado Variant	HG003	chr20	70,026	132	140	99.81%	99.80%	99.80%
		DeepVariant	HG003	chr20	70,077	86	89	99.88%	99.87%	99.88%
SNP	50x	LongcallD	HG003	chr20	70,007	225	159	99.68%	99.77%	99.73%
		Clair3 V2	HG003	chr20	6,696	547	3,932	92.44%	63.00%	74.93%
		Dorado Variant	HG003	chr20	5,535	3,502	5,093	61.23%	52.08%	56.29%
		DeepVariant	HG003	chr20	6,782	1,371	3,846	83.17%	63.81%	72.22%
	10x	LongcallD	HG003	chr20	6,729	10,749	3,899	39.10%	63.31%	48.34%
		Clair3 V2	HG003	chr20	7,753	347	2,875	95.71%	72.95%	82.79%
		Dorado Variant	HG003	chr20	7,924	1,560	2,704	83.54%	74.56%	78.79%
		DeepVariant	HG003	chr20	8,053	1,510	2,575	84.20%	75.77%	79.76%
	20x	LongcallD	HG003	chr20	7,840	7,088	2,788	53.22%	73.77%	61.83%
		Clair3 V2	HG003	chr20	8,249	332	2,379	96.13%	77.62%	85.89%
		Dorado Variant	HG003	chr20	8,542	1,388	2,086	86.01%	80.37%	83.09%
		DeepVariant	HG003	chr20	8,512	1,491	2,116	85.09%	80.09%	82.51%
	30x	LongcallD	HG003	chr20	8,100	5,900	2,528	58.58%	76.21%	66.24%
		Clair3 V2	HG003	chr20	8,516	382	2,112	95.70%	80.13%	87.23%
		Dorado Variant	HG003	chr20	8,861	1,212	1,767	87.96%	83.37%	85.60%
		DeepVariant	HG003	chr20	8,780	1,450	1,848	85.82%	82.61%	84.18%
	40x	LongcallD	HG003	chr20	8,263	5,457	2,365	60.96%	77.75%	68.34%
		Clair3 V2	HG003	chr20	8,786	390	1,842	95.75%	82.67%	88.73%
		Dorado Variant	HG003	chr20	8,968	1,148	1,660	88.64%	84.38%	86.46%
		DeepVariant	HG003	chr20	8,917	1,443	1,711	86.06%	83.90%	84.97%
Indel	50x	LongcallD	HG003	chr20	8,335	5,092	2,293	62.83%	78.42%	69.77%

Supplementary figures

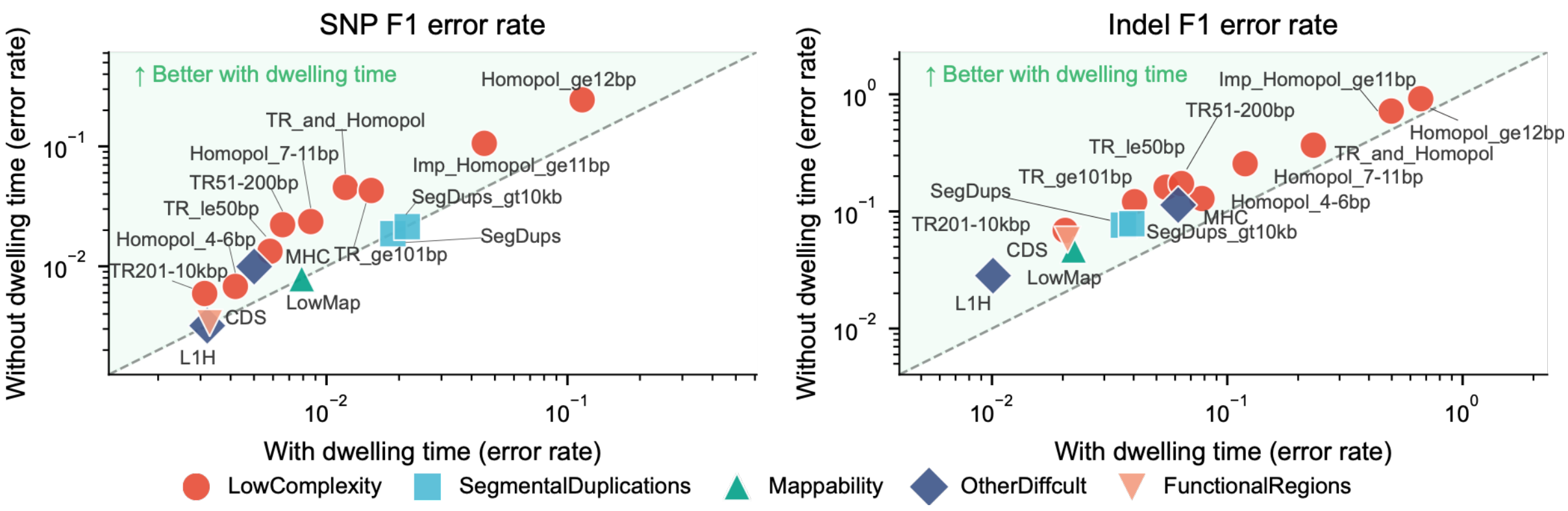

Supplementary Figure 1 Performance in complex genomic regions (HG001). *F1 error rates (1 – F1) for SNPs (left) and indels (right) across 16 stratified regions on HG001 at 30× coverage. Points above diagonal indicate improvement with Clair3 v2.*

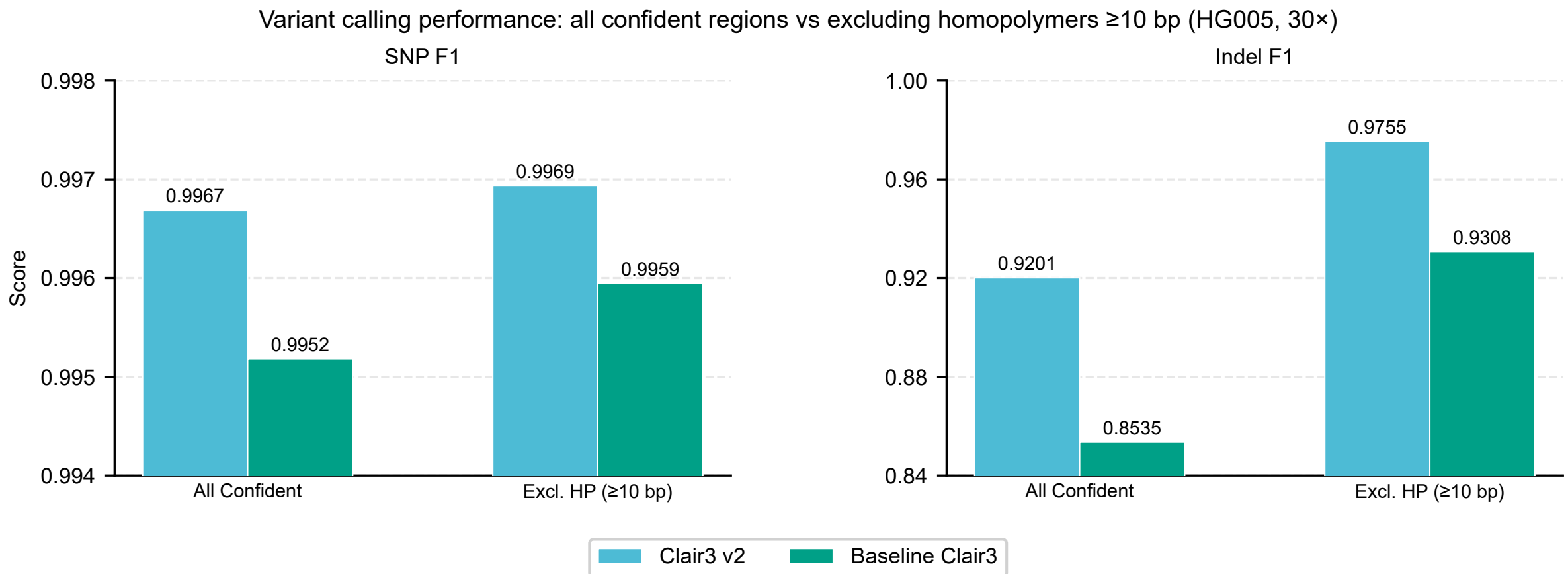

Supplementary Figure 2 Variant calling performance with and without homopolymer regions (HG005). *F1 scores for SNPs (left) and indels (right) evaluated in all confident regions and after excluding homopolymers ⩾10 bp on HG005 at 30× coverage.*
